# The 30th summer school of the Research Community for Mechanisms of Mutations

**DOI:** 10.1186/s41021-018-0093-4

**Published:** 2018-03-05

**Authors:** Takashi Yagi, Masanobu Kawanishi, Kazuhiko Takahashi

**Affiliations:** 1 Graduate School of Science, Osaka Prefecture University, 1-2 Gakuen-cho, Naka-ku, Sakai, Osaka, 599-8570 Japan; 2 Yokohama University of Pharmacy, Yokohama, Japan

**Keywords:** Mutagenesis, Carcinogenesis, Genotoxicity, The Research Community for Mechanisms of Mutations, The Japanese Environmental Mutagen Society

## Abstract

The 30th summer school of the Research Community for Mechanisms of Mutations was held on September 2nd-3rd, 2017 at the Kyoto Prefecture Seminar House. The Community celebrated the 30th anniversary of the school this year. The Community has been organizing a meeting once a year since it was founded as the Society for Mechanisms of Anti-mutagenesis and Anti-carcinogenesis Studies in 1987. The Society was reorganized to the current Community in 2006, and since then has a summer school aimed at providing information on mutation research frontiers and exchanging scientific information among young scientists such as graduate students, post-doctoral fellows, and assistant professors. This year, three eminent scientists were invited to discuss radiation cluster damage, the evolution of snake venom, and colibactin and colorectal cancer, while 15 participants presented their own research. Fifty-six participants joined in enthusiastic discussions and acquired new scientific knowledge.

## Background

The Japanese Environmental Mutagen Society (JEMS) aims to promote basic and applied research on environmental mutagens. JEMS research includes searches for unidentified mutagenic substances in foods, water and the atmosphere; the measurement of mutagenic substances in the environment; the elucidation of mutagenic and carcinogenic mechanisms; the elucidation of differences in genetic susceptibility among humans or animals; investigations on the complex effects of some mutagens; genotoxicity tests on foods, medicine and chemical materials; the development of and modifications to genotoxicity tests; and carcinogenic risk evaluations of substances [1, 2]. JEMS owns two official research groups for specific fields: the Bacterial Mutagenicity Study Group and Mammalian Mutagenicity Study Group. The Research Community for Mechanisms of Mutations (RCMM) is not owned by JEMS, but has a close relationship with JEMS in terms of membership and research interests [3]. Most governing board members of the RCMM belong to JEMS. The RCMM runs a summer school once a year on advanced topics related to mutagenesis, carcinogenesis, toxicity, pollution, and their associated subjects. Many university professors and institutional senior researchers bring their students to the school in order to accustom them to the research community, gain experience of presentations, and communicate with researchers in other laboratories. The summer school includes oral presentations by a few invited speakers and by many young participants, mainly graduate students, post-doctoral fellows, and assistant professors.

### History of the RCMM

The Japanese Society for Mechanisms of Anti-mutagenesis and Anti-carcinogenesis Studies (SMAAS), the former organization of the RCMM, was established in 1987. The SMAAS organized the first meeting as a satellite meeting of the 16th JEMS Annual Meeting in Kyoto by Prof. H. Nishioka, Doshisha University. The SMAAS became the main body to organize the 2nd International Conference on Mechanisms of Anti-mutagenesis and Anti-carcinogenesis held in Ohito, Japan in 1988 [4]. This international conference was directed by Dr. Y. Kuroda, National Institute of Genetics, and sponsored by two organizations, the JEMS and the Japanese Radiation Research Society. The contents of this international conference were summarized in a book [5]. The SMAAS organized domestic meetings on anti-mutagenesis and anti-carcinogenesis once a year until 2006, at which time the SMAAS was reorganized to the RCMM. At that time, the representative of the RCMM was switched from Prof. H. Nishioka to Dr. K. Takahashi, Nagoya City University, who is now Professor of Yokohama University of Pharmacy. The RCMM set a new aim to share recent advances in mutagenesis research among scientists and assist students to grow as scientists in this research field. The RCMM has been organizing a summer school every year. Many professors in the mutagenesis field also attend the summer school with their students because they were strongly influenced by the RCMM summer school when they were young researchers.

### Topics at the 30th summer school of the RCMM

**Fig. 1:**
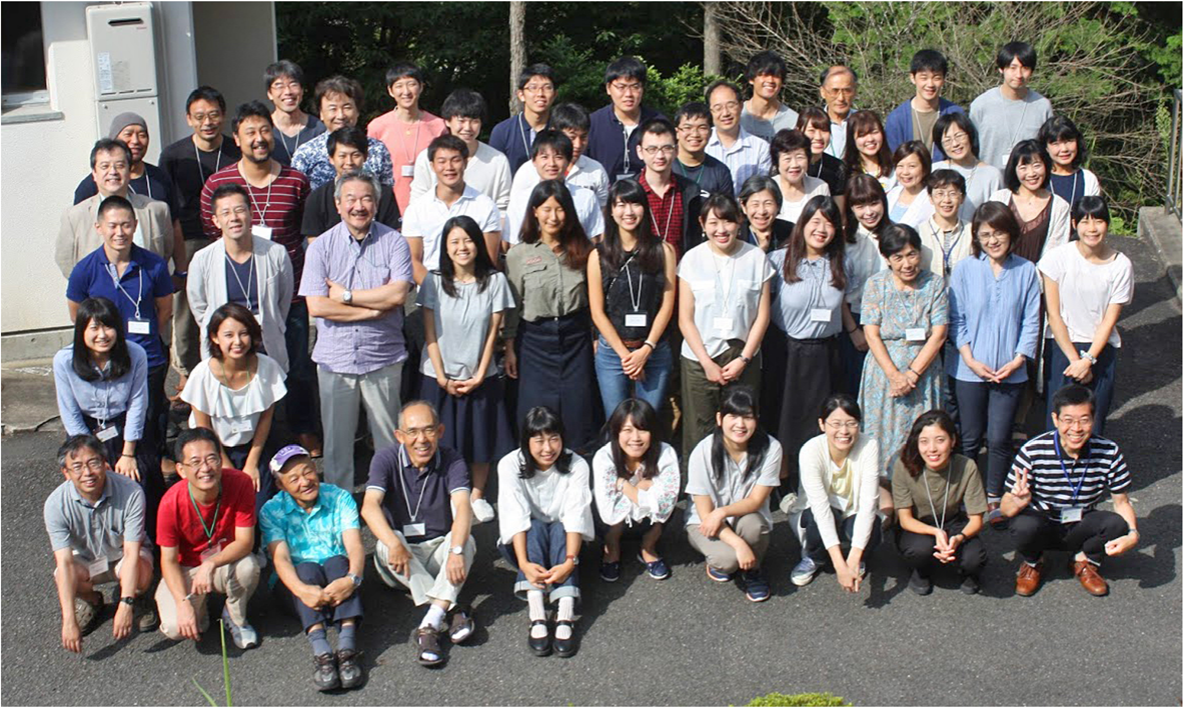
Participants of the 30th summer school of the RCMM

This year, the RCMM summer school was held on September 2nd-3rd, 2017 at the Kyoto Prefecture Seminar House, and was attended by 56 people (Fig. 1) [3]. Participants celebrated the 30th anniversary of the summer school since the Community was founded as the Society of Anti-mutagenesis and Anti-carcinogenesis Studies in 1987. Three invited scientists discussed their current research at the plenary session.

Dr. Narumi Shioi (Fukuoka University) presented “Poisonous snakes have defense mechanisms against their own venom –diversity of venoms and their inhibitory proteins evolved by unique mutations–”. The venoms of the Japanese poisonous snakes, *Protobothrops flavoviridis* and *Gloydius blomhoffii* (Viperidae) contain metalo protease, phospholipase A2, and serine protease. If vertebrates are bitten by these snakes, their venoms induce various disorders: bleeding due to the disruption of the blood vessel basement membrane; the inhibition of platelet aggregation; the decomposition of fibrinogen; and muscle necrosis. Dr. Shioi discovered the proteins that inhibit the enzyme activities of the respective venoms in the blood of the poisonous snakes. These proteins are called small serum proteins (SSPs) and habu serum factor (HSF). Five types of SSPs are expressed from 5 different paralogous genes. The coding sequences of these genes have higher frequencies of non-synonymous substitutions than those of the other genes, suggesting that this molecular diversity is obtained during the evolutionary processes of poisonous snakes [6, 7].

Dr. Kenji Watanabe (University of Shizuoka) presented “Analytical method of colibactin, a mutagen originated from intestinal bacterial flora, and the relationship between colibactin carrier and colorectal cancer in the Japanese cohort”. Colibactin is a low-molecular-weight compound secreted from some types of *Escherichia coli* and causes DNA double-strand breaks in mammalian cells and gastrointestinal cancer in mice. The chemical structure of colibactin has not yet been elucidated even though it was discovered more than 10 years ago [8, 9]. Dr. Watanabe found a precursor substance contained in the feces of those infected with enterotoxigenic *E. coli*. He designed and synthesized a probe molecule to conventionally detect colibactin without expensive analytical instruments. He also performed an epidemiological study to elucidate the relationship among lifestyle, colibactin infection, and colorectal cancer.

Dr. Ken Akamatsu (National Institutes for Quantum and Radiological Science and Technology) presented “Irreparable cluster DNA damage”. Cluster DNA damage involves multiple lesions formed at a small region of DNA, and is often induced by high LET radiation. The fluorescence resonance energy transfer (FRET) technique with two DNA-binding fluorescent molecules has been used to measure the extent of cluster DNA damage. One molecule is a fluorescence donor and the other is an acceptor molecule (hetero-FRET); however, this method does not have sufficiently high sensitivity or accessibility for measuring small amounts of damage. Dr. Akamatsu developed a homo-FRET technique using a single molecule with fluorescence anisotropy, and succeeded in measuring synthetic apyrimidinic/apurinic sites in a dose-dependent manner [10, 11].

## Conclusions

The 30th summer school of the RCMM held on September 2nd-3rd, 2017 finished successfully (Fig. 1). This year, the RCMM celebrated the 30th anniversary of the summer school and thanked those engaged in previous schools and meetings over the course of 30 years. Fifty-six people participated in the school and intensely discussed many topics. Three invited professors presented their latest research topics in the mutagenesis and toxicology fields, and 15 participants discussed their own research. Participants enjoyed hearing and discussing recent advances in mutagenesis research as well as communicating with researchers from other institutions. We hope to meet more people and have active discussions continuously at the summer school in the years ahead.

## Abbreviations

RCMM: The Research Community for Mechanisms of Mutations
FRET: Fluorescence resonance energy transfer
JEMS: The Japanese Environmental Mutagen Society
LET: Linear energy transfer
SMAAS: The Japanese Society for Mechanisms of Anti-mutagenesis and Anti-carcinogenesis Studies
SSPs: Small serum proteins
